# Comparative Evaluation of Radioiodine and Technetium-Labeled DARPin 9_29 for Radionuclide Molecular Imaging of HER2 Expression in Malignant Tumors

**DOI:** 10.1155/2018/6930425

**Published:** 2018-06-06

**Authors:** Anzhelika Vorobyeva, Olga Bragina, Mohamed Altai, Bogdan Mitran, Anna Orlova, Alexey Shulga, Galina Proshkina, Vladimir Chernov, Vladimir Tolmachev, Sergey Deyev

**Affiliations:** ^1^Department of Immunology, Genetics and Pathology, Uppsala University, Uppsala, Sweden; ^2^Nuclear Medicine Department, Cancer Research Institute, Tomsk National Research Medical Center Russian Academy of Sciences, Tomsk, Russia; ^3^Department of Medicinal Chemistry, Uppsala University, Uppsala, Sweden; ^4^Molecular Immunology Laboratory, Shemyakin & Ovchinnikov Institute of Bioorganic Chemistry, Russian Academy of Sciences, Moscow, Russia; ^5^National Research Tomsk Polytechnic University, Tomsk, Russia; ^6^Bio-Nanophotonic Lab., Institute of Engineering Physics for Biomedicine (PhysBio), National Research Nuclear University “MEPhI”, Moscow, Russia

## Abstract

High expression of human epidermal growth factor receptor 2 (HER2) in breast and gastroesophageal carcinomas is a predictive biomarker for treatment using HER2-targeted therapeutics (antibodies trastuzumab and pertuzumab, antibody-drug conjugate trastuzumab DM1, and tyrosine kinase inhibitor lapatinib). Radionuclide molecular imaging of HER2 expression might permit stratification of patients for HER2-targeting therapies. In this study, we evaluated a new HER2-imaging probe based on the designed ankyrin repeat protein (DARPin) 9_29. DARPin 9_29 was labeled with iodine-125 by direct radioiodination and with [^99m^Tc]Tc(CO)_3_ using the C-terminal hexahistidine tag. DARPin 9_29 preserved high specificity and affinity of binding to HER2-expressing cells after labeling. Uptake of [^125^I]I-DARPin 9_29 and [^99m^Tc]Tc(CO)_3_-DARPin 9_29 in HER2-positive SKOV-3 xenografts in mice at 6 h after injection was 3.4 ± 0.7 %ID/g and 2.9 ± 0.7 %ID/g, respectively. This was significantly (*p* < 0.00005) higher than the uptake of the same probes in HER2-negative Ramos lymphoma xenografts, 0.22 ± 0.09 %ID/g and 0.30 ± 0.05 %ID/g, respectively. Retention of [^125^I]I-DARPin 9_29 in the lung, liver, spleen, and kidneys was appreciably lower compared with [^99m^Tc]Tc(CO)_3_-DARPin 9_29, which resulted in significantly (*p* < 0.05) higher tumor-to-organ ratios. The biodistribution data were confirmed by SPECT/CT imaging. In conclusion, radioiodine is a preferable label for DARPin 9_29.

## 1. Introduction

Human epidermal growth factor 2 (HER2) is a tyrosine kinase receptor, which is overexpressed in about 15–20% of breast cancer [1] and 7–38% gastroesophageal cancers [2]. American Society of Clinical Oncology recommends HER2-targeted therapy for patients with HER2-positive metastatic breast cancer [3]. The use of anti-HER2 antibodies in combination with docetaxel in neoadjuvant setting also improves pathological response rate [4]. Since the overexpression of HER2 is a precondition for response to such therapies, College of American Pathologists (CAP), American Society for Clinical Pathology (ASCP), and the American Society of Clinical Oncology (ASCO) recommend assessment of HER2 status in all patients with breast and gastroesophageal carcinomas [3, 5]. Immunohistochemistry (IHC) and fluorescence in situ hybridization (FISH) assays are the recommended methods for HER2 molecular characterization of primary, recurrent, and metastatic tumors [5]. However, heterogeneity of HER2 expression remains a challenge. Discordances in HER2 expression between primary tumor and metastases are observed in up to 40% of the cases [6–8]. Additionally, the expression of HER2 might change over time leading to failure of HER2-targeted therapy.

Molecular imaging of HER2 expression is a noninvasive method that allows repeated whole-body monitoring of HER2 status [9]. Several types of targeting probes, such as monoclonal antibodies, their proteolytic and engineered fragments, and scaffold proteins, were evaluated for imaging of HER2 [9]. Comparison of data suggests that smaller targeting probes offer an advantage of providing good contrast and enabling imaging of HER2 at the day of injection. In addition, probes with a molecular weight of less than 45 kDa do not accumulate in tumors due to an enhanced permeability and retention (EPR) effect [10]. Thus, smaller targeting probes have a potential to improve both sensitivity and specificity of imaging.

One promising way of creating proteinaceous targeting probes for imaging is the use of engineered scaffold proteins (ESPs) [11–14]. ESPs are developed using a robust polypeptide framework (scaffold) found in natural proteins or designed de novo. Some amino acids in a scaffold are substituted by a random or directed mutagenesis, which generates big libraries and enables selection of specific high-affinity binders to designated molecular targets [12]. Several scaffolds, such as affibody molecules, knottins, designed ankyrin repeat proteins (DARPins), adnectins, and ADAPTs, have been preclinically evaluated for radionuclide molecular imaging and demonstrated capacity of imaging molecular targets in vivo at the day of injection [14]. Affibody molecules, which are based on the scaffold of protein A, have demonstrated that they can be used for in vivo quantification of HER2 expression in breast cancer metastases using PET and SPECT in the clinic [15, 16].

DARPins are built using a scaffold consisting of tightly packed repeats. Every repeat is a sequence of 33 amino acids formed as a *β*-turn followed by two antiparallel *α*-helices [17]. In a DARPin, two or three such repeats are flanked by C- and N-terminal capping repeats forming a protein with a molecular weight of 14–18 kDa. The use of ribosomal or phage display enables selection of binders with low nanomolar or subnanomolar affinity to predetermined targets. DARPins can be cost efficiently produced in prokaryotic hosts. Currently, DARPins are actively evaluated for different therapeutic applications [17].

Earlier, an anti-HER2 DARPin G3 was conjugated with a macrocyclic chelator DOTA, labeled with the radionuclide indium-111, and evaluated for imaging of HER2-expressing xenografts [18]. That study demonstrated the feasibility of using DARPins for radionuclide molecular imaging of HER2. Still, our experience with affibody-based imaging probes suggests that the composition of the binding site of a scaffold protein may influence off-target interaction of an imaging probe affecting its accumulation in normal tissues and blood clearance rate [19–21]. This may appreciably influence the imaging contrast and, therefore, sensitivity of diagnostics. We concluded that “clones of scaffold proteins should be evaluated to select the best variant for development of an imaging probe with optimal sensitivity for the intended application” [21]. This is in agreement with the findings of Zahnd et al. [22], who found appreciable difference in the blood clearance rate between variants of DARPin G3 with different mutations in the binding site. Therefore, we have selected another variant of the anti-HER2 DARPins, DARPin 9_29, to evaluate it as targeting agent for imaging of HER2 [23, 24].

**Figure 1:**
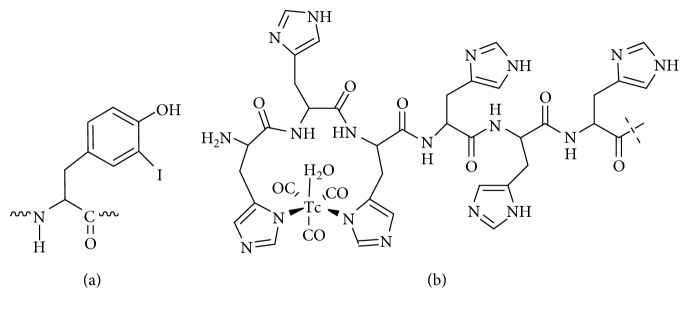
Structures of the radiolabeled moiety on the protein: iodine-125 label attached to tyrosine (a) and [^99m^Tc]Tc(CO)_3_ chelated by hexahistidine tag (b).

Selection of an optimal radionuclide is essential for development of a sensitive probe. Currently, SPECT/CT scanners are the most available imaging devices. Therefore, the use of a single photon emitter as a label might facilitate clinical translation of an imaging probe in hospitals where PET is not available due to logistic or economic reasons. Radionuclides technetium-99m (*T*_1/2_ = 6 h, E*γ* = 140 keV) and iodine-123 (*T*_1/2_ = 13.3 h, E*γ* = 159 keV) would offer several advantages for SPECT applications, such as optimal gamma-energies for imaging, short half-life, and low absorbed doses to patients. They permit the use of low energy collimators, providing higher resolution, minimizing the partial volume effect and increasing the registration efficiency. Direct radioiodination is a straightforward and robust method of radioiodination of targeting proteins. The use of tricarbonyl chemistry enables site-specific ^99m^Tc-labeling of histidine-tag-containing proteins using a kit technology [25]. Besides, a radioiodine label on a tyrosine (Figure 1(a)) is nonresidualizing while [^99m^Tc]Tc(CO)_3_ label on a histidine tag (Figure 1(b)) possesses strong residualizing properties. Evaluation of several labels might enable selection of a probe with optimal imaging properties due to differences in residualizing properties of radiocatabolites.

The goal of this study was to evaluate DARPin 9_29 labeled with radioisotopes of iodine and technetium as a targeting agent. The radioisotope [^125^I]I was used in this study as a surrogate label due to convenience. Labeling of DARPin 9_29 with iodine-125 and technetium-99m was established, the labeled proteins were evaluated in cell assays *in vitro*, and their biodistribution was compared directly *in vivo*. Specificity of HER2 targeting was evaluated in HER2-positive and HER2-negative breast cancer xenografts in mice.

## 2. Materials and Methods

### 2.1. General Materials and Instruments

Sodium iodide [^125^I]NaI was purchased from PerkinElmer Sverige AB (Sweden). Technetium-99m was obtained as pertechnetate by elution of Ultra-TechneKow generator (Mallinckrodt) with sterile 0.9% sodium chloride (Mallinckrodt, The Netherlands). The Center for Radiopharmaceutical Sciences (CRS) kits for production of tricarbonyl technetium were purchased from the CRS (PSI, Villigen, Switzerland). HPLC analysis was performed using Hitachi Chromaster HPLC systems with a radioactivity detector. The Vydac RP C18 protein column (300 Å, 3 × 150 mm, 5 *μ*m particle size) was used for the analysis. Solvent A was 0.1% trifluoroacetic acid in H_2_O, and solvent B was 0.1% trifluoroacetic acid in acetonitrile. The flow rate was 1 ml/min with a gradient of 5% B to 80% B over 20 minutes. Instant thin-layer chromatography (iTLC) analysis was performed using iTLC silica gel strips (Varian, Lake Forest, CA, USA). The distribution of activity was measured by a Cyclone storage phosphor system (Packard) and analyzed by OptiQuant image analysis software. Size-exclusion chromatography was performed with disposable NAP-5 columns (GE Healthcare). Activity was measured using an automated gamma-spectrometer with a NaI(TI) detector (1480 Wizard, Wallac, Finland). SKOV-3, BT474, DU145, and Ramos cells were purchased from the American Type Culture Collection (ATCC) and were cultured in complete RPMI medium supplemented with 10% fetal bovine serum (FBS), 2 mM L-glutamine, 100 IU/ml penicillin, and 100 *µ*g/ml streptomycin in a humidified incubator with 5% CO_2_ at 37°C, unless stated otherwise.

### 2.2. Production of DARPin 9_29

The gene for DARPin 9_29 was deduced from a DARPin 9_29 amino acid sequence deposited in PDB (Accession number PDB: 4HRL_A) taking into account the codon usage in highly expressed *E. coli* genes [26]. The gene was assembled from chemically synthesized overlapped oligonucleotides of 50 bp length by PCR and placed into pET22 plasmid vector between restriction sites NdeI and HindIII. The resultant amino acid sequence encoded by the gene was as follows: MDLGKKLLEAARAGQDDEVRILMANGADVNAHDFYGITPLHLAANFGHLEIVEVLLKHGADVNAFDYDNTPLHLAADAGHLEIVEVLLKYGADVNASDRDGHTPLHLAAREGHLEIVEVLLKNGADVNAQDKFGKTAFDISIDNGNEDLAEILQKLAAALEHHHHHH.

The DARPin 9_29 gene was expressed in *E. coli* strain BL21(DE3). Cells were grown overnight in the autoinduction ZYM-5052 medium [27] containing 100 *μ*g/ml ampicillin at 25°C. The cells were harvested by centrifugation at 10,000 g for 15 min at 4°C and resuspended in 1/6th of volume of lysis buffer (200 mM Tris-HCl, 500 mM sucrose, 1 mM EDTA, pH 8.0, 1 mM PMSF, and 60 *μ*g/ml lysozyme). The suspension was diluted two-fold with distilled water and incubated at room temperature for 30 min. Cells were broken on ice by a Vibra Cell ultrasonic liquid processor VCX130 (Sonics, USA) in a cycle mode of 10 s sonication, 10 s cooling, for a total of 30 cycles. The cellular debris was pelleted at 70,000 g for 60 min at 4°C. After addition of imidazole (30 mM final concentration) and NaCl (500 mM final concentration), the supernatant was filtered through a 0.22 *μ*m membrane and applied onto a HisTrap HP, 1 ml column (GE Healthcare) equilibrated with 20 mM sodium phosphate buffer, pH 7.5, 500 mM NaCl and 30 mM imidazole. Column was washed with ten volumes of the same buffer. The bound protein was eluted with a linear 30–500 *μ*M imidazole gradient. The fractions containing DARPin 9_29 were pooled and desalted on the PD10 column (GE Healthcare). The protein solution was loaded onto a MonoQ 5/50 GL column (GE Healthcare, USA) equilibrated with 20 mM Tris-HCl, pH 8.0. After washing the column with the same buffer, the bound protein was eluted with a linear 0-1 M NaCl gradient. The fractions were analyzed by 15% reducing SDS-PAGE. The fractions containing DARPin 9_29 were pooled and concentrated with a Amicon Ultra-15 centrifugal filter and then sterilized by filtration through 0.22 *μ*m membrane. Protein concentration was determined by UV spectroscopy using *ɛ*280 = 4470 cm^−1^·M^−1^ [28]. No proteinaceous impurities were found using HPLC analysis.

### 2.3. Labeling and Stability

Direct radioiodination was performed as described previously [29]. To a solution of DARPin 9_29 (40 *µ*g, 2.20 nmol) in PBS (47 *µ*L), [^125^I]NaI (3 *µ*L, 10 MBq) and chloramine T (20 *µ*L of 1 mg/mL in PBS, 20 *µ*g, 71 nmol) were added. After incubation at room temperature for 60 sec, sodium metabisulfite (20 *µ*L of 2 mg/mL in water, 40 *µ*g, 211 nmol) was added. The reaction yield was analyzed by iTLC, and the radiolabeled compound was purified using NAP-5 size-exclusion column (preequilibrated with 1% BSA in PBS) and eluted with PBS. The *in vitro* stability test was performed by incubating the radioiodinated protein with 1 M KI in PBS at room temperature for 3 h. Control samples (without KI) were incubated in PBS. Both sample groups were analyzed by iTLC.

Site-specific radiolabeling of DARPin 9_29 with [^99m^Tc][Tc(CO)_3_(H_2_O)_3_]^+^ was performed as described earlier [30]. The [^99m^Tc]NaTcO_4_ eluate (400–500 *μ*L) containing ca. 4 GBq of ^99m^Tc was added to a sealed vial containing CRS kit and incubated at 100°C for 20 min. After incubation, 40 *μ*L of technetium-99m tricarbonyl solution was added to a tube containing 168 *μ*g of DARPin 9_29 in 100 *μ*L of PBS. The reaction was incubated for 60 min at 40°C, and then, the radiolabeled DARPin 9_29 was purified using NAP-5 columns preequilibrated and eluted with PBS. The *in vitro* stability test was performed by incubating the radiolabeled protein with 500- and 5000-fold molar excess of histidine in PBS for 3 h. Control samples were incubated in PBS, and both sample groups were analyzed by iTLC.

The radiochemical yield and radiochemical purity after NAP-5 purification were determined by iTLC. Radio-iTLC analysis was performed in 4 : 1 acetone: water system and in PBS for DARPin 9_29 labeled with [^125^I]I and [^99m^Tc]Tc(CO)_3_, respectively. In both systems, the radiolabeled DARPins remain at the application point, and all forms of free radionuclides (including [^99m^Tc]TcO_4_^−^ and [^99m^Tc][Tc(CO)_3_(H_2_O)_3_]^+^) migrate with the solvent front.

### 2.4. In Vitro Binding Specificity and Cellular Processing

In vitro studies were performed using HER2-expressing cell lines SKOV-3 (1.6 × 10^6^ receptors/cell) [19], BT474 (1.2 × 10^6^ receptors/cell) [31], and DU145 (5 × 10^4^ receptors/cell) [32]. Cells were seeded in 3 cm Petri dishes (10^6^ cells per dish), and a set of three dishes was used for each group.

HER2 binding specificity assay was performed as described previously [33]. Two sets of dishes were used per each cell line. A 100-fold excess of unlabeled DARPin 9_29, cetuximab, trastuzumab, bevacizumab, or anti-HER2 affibody molecule Z_HER2:342_ (1000 nM) was added to the control group of cells to saturate HER2 receptors 30 min before addition of the labeled compound. Radiolabeled conjugates [^125^I]I-DARPin 9_29 or [^99m^Tc]Tc(CO)_3_-DARPin 9_29 were added at 10 nM concentrations. The cells were incubated for 1 h in a humidified incubator at 37°C. The medium was collected, the cells were washed with 1 mL of fresh medium. and 1 mL 1 M NaOH was added to lyse the cells. After 20–30 min of incubation, the cell lysate was collected. The activity in each fraction was measured to calculate the percent of cell-bound activity. The average number of cells per dish at the time of assay was calculated, and the value of cell-bound activity was calculated per 10^6^ cells.

Cellular retention and processing of radiolabeled conjugates by SKOV-3 and BT474 cells was studied during continuous incubation by an acid-wash method [33]. The cells (1 × 10^6^ cells/dish) were seeded two days before the experiment (three dishes for each time point). Radiolabeled [^125^I]I-DARPin 9_29 or [^99m^Tc]Tc(CO)_3_-DARPin 9_29 (10 nM) was added to all cells and incubated at 37°C in a humidified incubator. At 1, 2, 4, 6, and 24 h postaddition, the medium was collected from one set of 3 dishes and cells were washed once with serum-free media (1 mL). To collect the membrane-bound tracer, the cells were treated with 0.2 M glycine buffer containing 4 M urea, pH 2.0 (1 mL) on ice for 5 min, the buffer was collected, and the cells were washed once with 1 mL of buffer. To lyse the cells containing internalized conjugate, the cells were treated with 1 M NaOH (1 mL) for 30 min, and the cells were collected and additionally washed with 1 mL. The activity in the acid fractions was considered as membrane bound, in the alkaline fractions, as internalized.

### 2.5. Affinity Measurement Using LigandTracer

The binding kinetics of radiolabeled DARPins to living SKOV-3 cells was measured using LigandTracer (Ridgeview Instruments, Vänge, Sweden) as described previously [29]. Kinetics of binding to and dissociation from living cells was recorded at room temperature in real time. The TraceDrawer Software (Ridgeview Instruments, Vänge, Sweden) was used to calculate the affinity based on the association and dissociation rates. Increasing concentrations of each radioconjugate (1, 4, and 8 nM) were added to the cells followed by the change of media and measurements of retention in the dissociation phase.

### 2.6. Animal Studies

For tumor implantation, 10^7^ HER2-positive SKOV-3 cells or 5 × 10^6^ HER2-negative Ramos cells in 100 *µ*L of media were subcutaneously injected on the right hind leg of female BALB/c nu/nu mice. The biodistribution experiments were performed two and a half weeks after cell implantation. The average animal weight was 16 ± 1 g in the SKOV-3 group and 17 ± 1 g in the Ramos group. The average tumor weight was 0.26 ± 0.16 g for SKOV-3 xenografts and 0.22 ± 0.14 g for Ramos xenografts. Twenty-four hours before the experiments drinking water was replaced with 1% KI solution in water. For comparative biodistribution of [^125^I]I-DARPin 9_29 and [^99m^Tc]Tc(CO)_3_-DARPin 9_29, a dual-isotope approach was used. The mice were intravenously (i.v.) injected with a mixture of [^125^I]I-DARPin 9_29 and [^99m^Tc]Tc(CO)_3_-DARPin 9_29 (4 *μ*g in 100 *μ*L of 1% BSA in PBS/mouse, 15 kBq for [^125^I]I-DARPin 9_29, and 25 kBq for [^99m^Tc]Tc(CO)_3_-DARPin 9_29). At 6 h postinjection (p.i.), mice were anesthetized by an intraperitoneal injection of Ketalar and Rompun solution and sacrificed by heart puncture. Blood was collected with a heparinized syringe, and organs were collected and weighed, and activity was measured on a gamma-spectrometer using a dual-isotope protocol. The measurements were corrected for dead time, spillover, and background. The percent of injected dose per gram of sample (%ID/g) was calculated. Statistical analysis was performed using GraphPad Prism (GraphPad Software Inc.).

Whole-body SPECT/CT scans of the mice bearing SKOV3 xenografts injected with [^99m^Tc]Tc-DARPin 9_29 (4 *µ*g, 4.3 MBq) or [^125^I]I-DARPin 9_29 (4 *µ*g, 4.4 MBq) were performed using nanoScan SPECT/CT (Mediso Medical Imaging Systems, Hungary) at 6 h p.i. Imaging was performed under sevoflurane anesthesia. CT scans were acquired at the following parameters: 50  keV, 670  *μ*A, 480 projections, 5 min acquisition time; SPECT scans were carried out using technetium-99m energy window (126.45  keV–154.56  keV) or iodine-125 energy window (25.56  keV–31.24  keV), 256  ×  256 matrix, 30 min acquisition time. The CT raw data were reconstructed using Nucline 2.03 Software (Mediso Medical Imaging Systems, Hungary). SPECT raw data were reconstructed using Tera-Tomo™ 3D SPECT.

## 3. Results

### 3.1. Radiolabeling

Direct radioiodination of DARPin 9_29 provided a radiochemical yield of 96.2 ± 0.7%. Size-exclusion chromatography using disposable NAP-5 column provided radiochemical purity of 99.7 ± 0.5%. The isolated yield was 87 ± 1%. Specific activity of 1.1 MBq/µg (20 GBq/µmol) was achieved. Incubation of [^125^I]I-DARPin 9_29 in PBS or challenge with 1 M potassium iodide up to 3 h did not reveal any measurable release of the radionuclide from DARPin 9_29 (Table 1).

Labeling of DARPin 9_29 with [^99m^Tc]Tc(CO)_3_ resulted in a radiochemical yield of 80 ± 4%. The radiochemical purity after size-exclusion chromatography was 98 ± 1%. The isolated yield was 72 ± 8%. The maximum specific activity was 1.3 MBq/*µ*g (23.6 GBq/*µ*mol). The labeled protein was stable to a significant degree of up to 3 h in PBS and in both 500-fold and 5000-fold molar excess of histidine (Table 2).

### 3.2. In Vitro Evaluation of Radiolabeled DARPins

**Figure 2:**
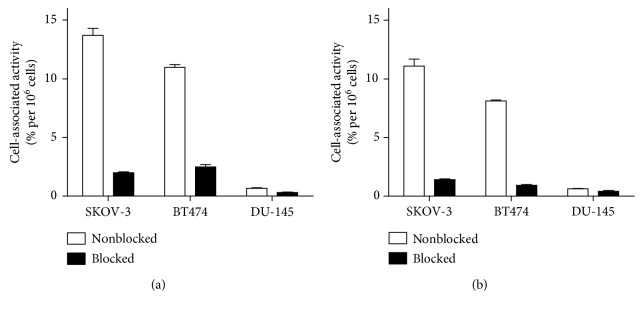
In vitro binding specificity of [^99m^Tc]Tc(CO)_3_-DARPin (a) and [^125^I]I-DARPin 9_29 (b) to HER2-expressing cells. In the blocked group, receptors were presaturated with a 100-fold excess of unlabeled DARPin. Data are presented as the mean of three samples ± SD.

Binding of both [^99m^Tc]Tc(CO)_3_-DARPin and [^125^I]I-DARPin 9_29 to HER2-expressing cells in vitro was dependent on the HER2 expression level in these cell lines (SKOV-3 > BT474 >> DU-145) (Figure 2). Blocking the receptors with a large molar excess of unlabeled DARPin 9_29 resulted in significant (*p* < 0.05) reduction of binding. This demonstrates binding saturability and suggests the specific binding character for both conjugates.

**Figure 3:**
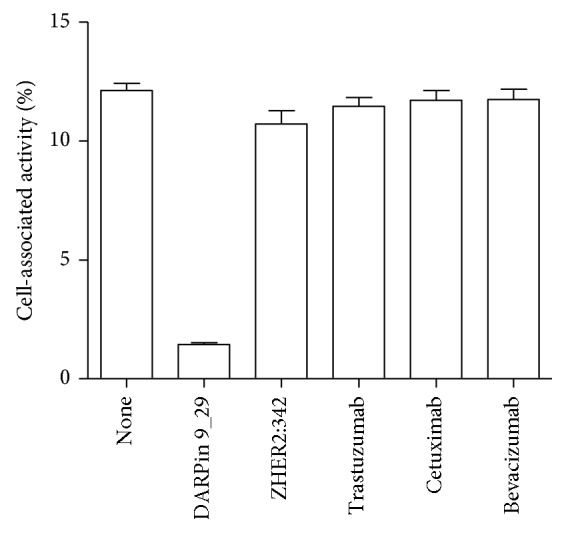
In vitro binding of [^125^I]I-DARPin 9_29 to SKOV-3 cells after treatment with 100-fold molar excess of different targeting agents. Data are presented as the mean of three samples ± SD.

To elucidate the binding specificity further, binding of [^125^I]I-DARPin 9_29 was determined after treatment of SKOV-3 cells with anti-HER2 affibody molecules Z_HER2:342_, anti-HER2 antibody trastuzumab, as well as control antibodies anti-EGFR cetuximab and anti-VEGF bevacizumab. As shown in Figure 3, while reduction in binding after treatment with unlabeled DARPin 9_29 was highly significant (*p* < 0.000001), there was neither significant reduction in binding after receptor saturation using trastuzumab (*p* > 0.05) nor any reduction in binding after treatment of SKOV-3 cells with cetuximab or bevacizumab. Interestingly, treatment of the SKOV-3 cells with Z_HER2:342_ affibody molecule resulted in small (12%) but significant (*p* < 0.05) reduction of [^125^I]I-DARPin 9_29 binding.

**Figure 4:**
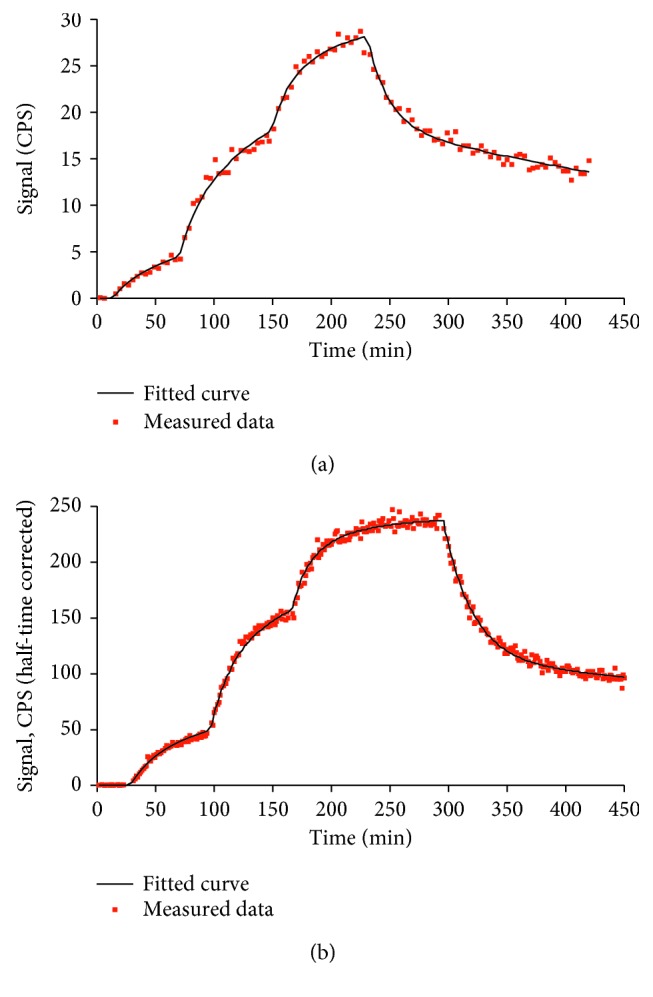
Representative LigandTracer sensorgrams of [^125^I]I-DARPin 9_29 (a) and [^99m^Tc]Tc(CO)_3_-DARPin 9_29 (b) binding to HER2-expressing SKOV-3 cells. The association was measured at concentrations of 1, 4, and 8 nM.

Representative LigandTracer sensorgrams of [^99m^Tc]Tc(CO)_3_-DARPin 9_29 and [^125^I]I-DARPin 9_29 binding to HER2-expressing SKOV-3 cells are presented in Figure 4. Sensorgrams show rapid binding and slow dissociation of the conjugates. The best curve fitting for both variants was obtained when 1 : 2 binding model was applied. The analysis suggested the presence of one interaction with high affinity and one with lower. The equilibrium dissociation constant (*K*_D_) of the strongest interaction was 362 ± 130 pM for [^125^I]I-DARPin 9_29 and 439 ± 156 pM for [^99m^Tc]Tc(CO)_3_-DARPin. In the case of weaker interactions, the dissociation constants were 7.7 ± 2.8 nM and 9.1 ± 2.7 nM for [^125^I]I-DARPin 9_29 and [^99m^Tc]Tc(CO)_3_-DARPin, respectively. The weaker interaction was approximately 3-fold more abundant.

**Figure 5:**
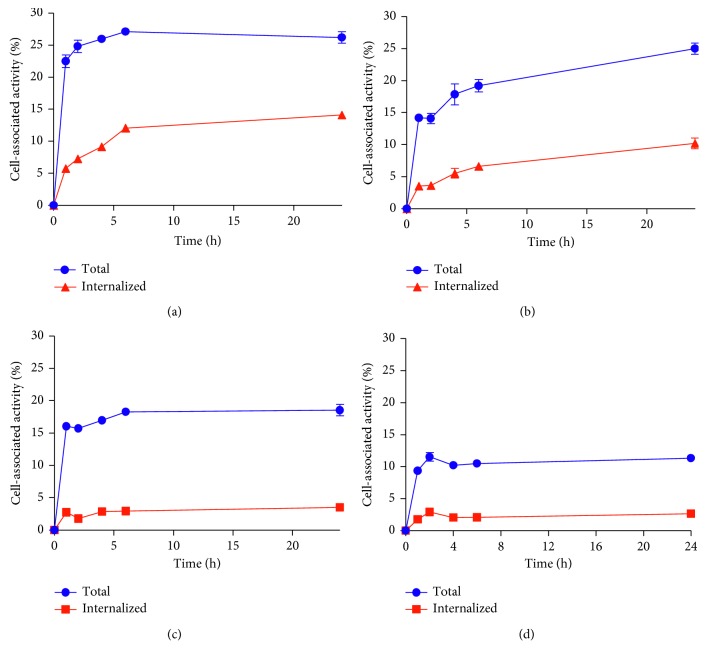
Cellular processing of [^99m^Tc]Tc(CO)_3_-DARPin 9_29 (a, b) and [^125^I]I-DARPin 9_29 (c, d) by HER2-expressing SKOV-3 (a, c) and BT474 (b, d) cells. Cells were incubated with the conjugates (10 nM) at 37°C. Data are presented as the mean of three samples ± SD.

Processing of [^99m^Tc]Tc(CO)_3_-DARPin and [^125^I]I-DARPin 9_29 by HER2-expressing SKOV-3 and BT474 cells during continuous incubation is presented in Figure 5. Pattern of the cellular processing was concordant in both cell lines. The internalized activity was lower for DARPin 9_29 labeled using nonresidualizing iodine-125. Total cell-associated activity was also lower in the case of [^125^I]I-DARPin 9_29. However, the internalized fraction was not very high (approximately 20–25% at 4 h) even for the residualizing [^99m^Tc]Tc(CO)_3_ label.

### 3.3. Animal Studies

**Figure 6:**
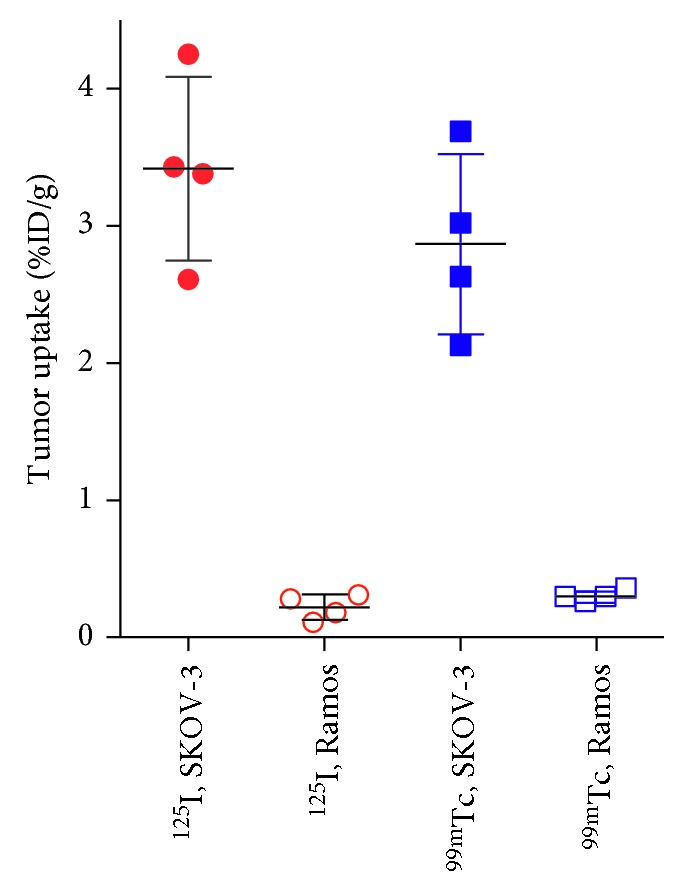
In vivo specificity of HER2 targeting using [^125^I]I-DARPin 9_29 and [^99m^Tc]Tc(CO)_3_-DARPin 9_29. Uptake of both imaging probes was significantly (*p* < 0.00005) higher in HER2-positive SKOV-3 than in HER2-negative Ramos xenografts. Data are presented as mean ± SD for four mice.

The results of the in vivo specificity test are presented in Figure 6. Accumulation of both [^125^I]I-DARPin 9_29 and [^99m^Tc]Tc(CO)_3_-DARPin 9_29 in HER2-negative Ramos lymphoma xenografts were on the same level as in muscles (0.2–0.3 %ID/g). Uptake in HER2-positive SKOV-3 xenografts was significantly (*p* < 0.00005) higher, 3.4 ± 0.7 %ID/g and 2.9 ± 0.7 %ID/g for [^125^I]I-DARPin 9_29 and [^99m^Tc]Tc(CO)_3_-DARPin 9_29, respectively. This demonstrates that the tumor uptake of both variants was HER2 specific.

Data concerning biodistribution of [^125^I]I-DARPin 9_29 and [^99m^Tc]Tc(CO)_3_-DARPin 9_29 in BALB/C nu/nu mice bearing HER2-positive xenografts at 6 h after injection are presented in Table 3. The common feature of both constructs was relatively rapid clearance from blood. However, there were appreciable differences in biodistribution of these variants. For example, [^125^I]I-DARPin 9_29 had significantly lower uptake in the lung, liver, spleen, kidneys, and bone. Besides, accumulation of radioiodine in the content of gastrointestinal tract was also lower, indicating that hepatobiliary excretion plays smaller role for this probe and/or its radiometabolites. There was no significant difference between tumor uptakes of [^125^I]I-DARPin 9_29 and [^99m^Tc]Tc(CO)_3_-DARPin 9_29.

Such biodistribution features were translated in tumor-to-organ ratios (Table 4). DARPin 9_29 labeled with iodine-125 provided significantly higher tumor-to-lung, tumor-to-liver, tumor-to-spleen, and tumor-to-bone ratios. Importantly, tumor-to-lung and tumor-to-liver ratio were more than one for the radioiodine label, enabling imaging in these metastatic sites for many malignancies.

**Figure 7:**
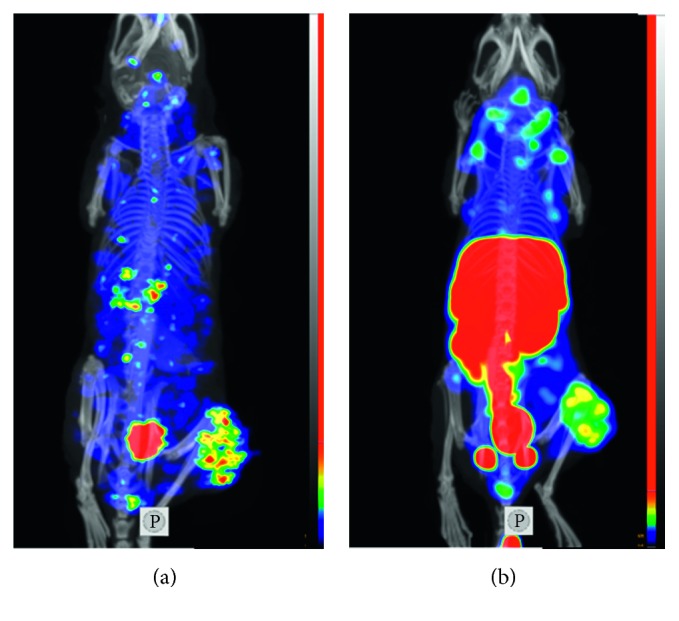
Imaging of HER2 expression in SKOV-3 xenografts (maximum intensity projection) using [^125^I]I-DARPin 9_29 (a) and [^99m^Tc]Tc(CO)_3_-DARPin 9_29 (b). Small-animal SPECT/CT imaging was performed at 6 h after injection.

The results of the small-animal SPECT/CT imaging (Figure 7) confirmed the results of the ex vivo biodistribution measurements. The HER2-expressing xenografts were well visualized using both [^125^I]I-DARPin 9_29 and [^99m^Tc]Tc(CO)_3_-DARPin 9_29. Due to renal excretion, an appreciable amount of activity was detected in the urinary bladder. In the case of [^99m^Tc]Tc(CO)_3_-DARPin 9_29, a noticeable amount of activity was detected in the gastrointestinal tract, liver, and kidneys. Activity concentration in these organs exceeded concentration in tumors. In the case of [^125^I]I-DARPin 9_29, the opposite was observed as the tumor had the highest activity accumulation (after the urinary bladder).

## 4. Discussion

We have demonstrated that DARPin 9_29 can be labeled with [^125^I]I and [^99m^Tc]Tc(CO)_3_ with reasonably good yields and high stability (Tables 1 and 2). Both labeled proteins retained specific binding to HER2-expressing cells, and the level of binding was proportional to HER2 expression in these cell lines (Figures 2 and 3). The binding of [^125^I]I-DARPin 9_29 could be saturated by nonlabeled DARPin 9_29 but not with monoclonal antibody trastuzumab (Figure 3). This is in agreement with the literature data suggesting that DARPin 9_29 and trastuzumab bind to different epitopes on HER2 [23]. This opens an opportunity to use radiolabeled DARPin 9_29 for monitoring of HER2 during trastuzumab therapy without interference from antibodies bound to the receptors on malignant cells. It was surprising that small but significant reduction of [^125^I]I-DARPin 9_29 binding to SKOV-3 cells was found after treatment of cells with a large excess of anti-HER2 affibody molecules Z_HER2:342_, although this affibody molecules bind to a different epitope [23]. A possible explanation is that the binding of affibody molecules causes some conformational change of the receptor, which is not favorable for DARPin 9_29 binding.

The LigandTracer measurements demonstrated that both labeled variants bind with similar affinity to SKOV-3 cells, with *K*_D1_ of approximately 0.4 nM and *K*_D2_ of 8-9 nM. Two affinities are often found during LigandTracer measurement of binding to living cells for tracers targeting receptors belonging to HER family [34–37]. Björkelund et al. [35] demonstrated that this can be explained by homo- and heterodimerization of receptors. The dissociation constant values are in a good agreement with that obtained earlier for binding of DARPin 9_29 to the extracellular domain of HER2 (3.8 nM) using surface plasmon resonance (SPR) [38]. The affinity measurements suggest that both methods provide labeled DARPin 9_29 with preservation of binding affinity to HER2.

The data concerning cellular processing of radiolabeled conjugates after binding to HER2-expressing cells (Figure 5) demonstrated a potential advantage of the residualizing ^99m^Tc(CO)_3_ label. The intracellular fraction was higher for technetium-99m compared to radioiodine, which translated into higher total cell-associated activity. However, the internalization was relatively slow, and the difference between total cellular uptake of [^99m^Tc]Tc(CO)_3_-DARPin 9_29 and [^125^I]I-DARPin 9_29 was moderate (1.5–1.7 fold) at 4 and 6 h, that is, time points relevant for imaging.

The uptake of both [^125^I]I-DARPin 9_29 and [^99m^Tc]Tc(CO)_3_-DARPin 9_29 in HER2-expressing SKOV-3 xenografts was significantly higher than in HER2-negative Ramos lymphoma xenografts, which is a strong evidence of HER2-specific tumor accumulation. For comparison, an EPR-mediated nonspecific uptake of anti-HER2 monoclonal antibodies might amount to 25–30% of specific one up to 3 days after injection [31, 39], which might be associated with an elevated risk of false-positive diagnosis.

There was a striking difference between the biodistribution profiles of [^125^I]I-DARPin 9_29 and [^99m^Tc]Tc(CO)_3_-DARPin 9_29 (Table 3 and Figure 7). Uptake of [^99m^Tc]Tc(CO)_3_-DARPin 9_29 in the lung, liver, spleen, and kidneys was several-fold higher than the uptake of [^125^I]I-DARPin 9_29. The high renal uptake is characteristic for radiometal-labeled DARPins [18, 22] and is, most likely, associated with the high reabsorption of proteins in proximal tubuli, rapid internalization, and efficient retention of radiometabolites of residualizing radiometal labels. The high hepatic uptake might be due to high lipophilicity of [^99m^Tc]Tc(CO)_3_-histidine label [40–42]. This effect depends on properties of a targeting protein. For example, elevated hepatic uptake in the case of [^99m^Tc]Tc(CO)_3_-histidine label was observed for DARPin G3 [22] and affibody molecule Z_HER2:342_ [43] but not observed for scFv [25], nanobodies [44], or ADAPTs [45]. The low accumulation of [^125^I]I in these organs is due to nonresidualizing properties of direct radioiodine label. Due to rapid internalization after renal and hepatic uptake, [^125^I]I-DARPin 9_29 undergoes lysosomal degradation, and the main radiometabolite, iodotyrosine, “leaks” from cells. Such effect has been described for affibody molecules [30] and ADAPTs [36]. The tumor uptake in vivo of [^125^I]I-DARPin 9_29 and [^99m^Tc]Tc(CO)_3_-DARPin 9_29 was not different because the internalization of DARPin 9_29 after binding to the HER2 receptor in cancer cells was slow, and leakage of radiometabolites had no strong effect on tumor-associated activity. In addition, the delivery of [^99m^Tc]Tc(CO)_3_-DARPin 9_29 to tumors might be reduced due to sequestering of this conjugate in the liver. The level of the tumor uptake, 3.4 ± 0.7 %ID/g and 2.9 ± 0.7 %ID/g for [^125^I]I-DARPin 9_29 and [^99m^Tc]Tc(CO)_3_-DARPin 9_29, respectively, was similar to the uptake of [^99m^Tc]Tc(CO)_3_-labeled DARPin G3-D (4.45 ± 1.18 %ID/g) with the similar affinity to HER2 D (1.48 ± 0.01 nM according to SPR measurements) [22]. The [^99m^Tc]Tc(CO)_3_-labeled anti-HER2 nanobody 2Rs15d having similar size and affinity (3.9 nM) had also comparable tumor uptake (4.19 ± 0.47 %ID/g) [44]. It has to be noted that both the nanobody and DARPin G3-D cleared more rapidly from blood and normal tissues, which resulted in higher tumor-to-organ ratios compared to DARPin 9_29 [22, 44]. Overall, the iodine-125 label provided better imaging contrast compared to the technetium-99m label. The use of technetium-99m might be limited to special applications, like determination of HER2 status in primary tumor and local lymph node metastases before neoadjuvant therapy of breast carcinomas.

This study utilized iodine-125 (*T*_1/2_ = 59.4 d) because this radionuclide is convenient for preclinical development due to long half-life and low energy of emitted photons. Our experience suggests that the labeling protocol might be applied with a minimal reoptimization for labeling with iodine-123 (*T*_1/2_ = 13.3 h) and iodine-124 (*T*_1/2_ = 4.18 d) for clinical SPECT or PET applications, respectively [46, 47].

## 5. Conclusion

DARPin 9_29 can be labeled with iodine-125 and [^99m^Tc]Tc(CO)_3_ with high stability and preserved binding specificity and affinity to HER2. Both labeled proteins were internalized slowly by HER2-expressing cells. Both [^125^I]I-DARPin 9_29 and [^99m^Tc]Tc(CO)_3_-DARPin 9_29 demonstrated specific uptake in HER2-positive xenografts when compared to HER2-negative xenografts. Radioiodine provided better tumor-to-organ ratios compared to [^99m^Tc]Tc(CO)_3_ label. Radioiodinated DARPin 9_29 is a promising agent for same-day imaging of HER2 expression in cancer using SPECT.

## Figures and Tables

**Table 1:** Stability of [^125^I]I-DARPin 9_29.

Test solution	DARPin-associated activity (%)	
	1 h	3 h
PBS	99.7 ± 0.4	97.9 ± 1.0
1 M KI	99.0 ± 0.3	98.5 ± 0.4

**Table 2:** Stability of [^99m^Tc]Tc(CO)_3_-DARPin 9_29.

Test solution	DARPin-associated activity (%)	
	1 h	3 h
PBS	99.8 ± 0.2	98.2 ± 0.1
Histidine (500-fold excess)	99.7 ± 0.4	98.7 ± 0.4
Histidine (5000-fold excess)	99.0 ± 0.3	97.7 ± 0.6

**Table 3:** Comparison of biodistribution of [^125^I]I-DARPin 9_29 and [^99m^Tc]Tc(CO)_3_-DARPin 9_29 in BALB/C nu/nu mice bearing SKOV-3 xenografts at 6 h after injection.

	Uptake (%ID/g)	
	^125^I-DARPin 9_29	^99m^Tc(CO)_3_-DARPin 9_29
Blood	1.2 ± 0.4	0.8 ± 0.1
Salivary glands	1.3 ± 0.3	1.7 ± 0.4
Lung	0.9 ± 0.3^*∗*^	1.7 ± 0.2
Liver	1.2 ± 0.2^*∗*^	27 ± 4
Spleen	0.9 ± 0.2^*∗*^	8.4 ± 3.0
Stomach	4.3 ± 3.0	1.3 ± 0.1
Kidney	3.4 ± 0.4^*∗*^	80 ± 11
Tumor	3.4 ± 0.7	2.9 ± 0.7
Muscle	0.3 ± 0.1	0.5 ± 0.1
Bone	0.77 ± 0.04^*∗*^	2.0 ± 0.8
Gastrointestinal tract^a^	1.4 ± 0.3^*∗*^	5.3 ± 0.9

^a^Data for the gastrointestinal tract are presented as %ID per whole sample.  ^*∗*^Significant difference (*p* < 0.05) between uptake of [^125^I]I-DARPin 9_29 and [^99m^Tc]Tc(CO)_3_-DARPin 9_29. Data are presented as mean ± SD for four mice.

**Table 4:** Comparison of [^125^I]I-DARPin 9_29 and [^99m^Tc]Tc(CO)_3_- DARPin 9_29 tumor-to-organ ratios in nude mice bearing SKOV-3 xenografts.

	Tumor-to-organ ratio	
	[^125^I]I-DARPin 9_29	[^99m^Tc]Tc(CO)_3_-DARPin 9_29
Blood	3.4 ± 1.8	3.4 ± 0.5
Salivary glands	2.0 ± 0.7	2.0 ± 0.4
Lung	4 ± 2	1.7 ± 0.4^*∗*^
Liver	3 ± 1	0.11 ± 0.02∗
Spleen	4 ± 1	0.35 ± 0.04^*∗*^
Stomach	1.2 ± 1.0	2.1 ± 0.5^*∗*^
Kidney	1.00 ± 0.16	0.035 ± 0.003^*∗*^
Muscle	11.3 ± 5.5	6.6 ± 2.6
Bone	4.4 ± 0.9	1.6 ± 0.4^*∗*^

^*∗*^Significant difference (*p* < 0.05) between values for [^125^I]I-DARPin 9_29 and [^99m^Tc]Tc(CO)_3_-DARPin 9_29. Data are presented as mean ± SD for four mice.

## Data Availability

Data supporting conclusions of this study are included in the article. Raw data are available from the corresponding author upon request.
